# Morphological Characterization, Polyphenolic Profile, and Bioactive Properties of *Limoncella*, an Ancient Mediterranean Variety of Sweet Citrus

**DOI:** 10.3390/biom14101275

**Published:** 2024-10-10

**Authors:** Lucia Potenza, Roberta Saltarelli, Francesco Palma, Laura Di Patria, Giosuè Annibalini, Sabrina Burattini, Pietro Gobbi, Laura Valentini, Giovanni Caprioli, Agnese Santanatoglia, Sauro Vittori, Elena Barbieri

**Affiliations:** 1 Department of Biomolecular Sciences, University of Urbino Carlo Bo, 61029 Urbino, Italy; lucia.potenza@uniurb.it (L.P.); roberta.saltarelli@uniurb.it (R.S.); giosue.annibalini@uniurb.it (G.A.); sabrina.burattini@uniurb.it (S.B.); pietro.gobbi@uniurb.it (P.G.); laura.valentini@uniurb.it (L.V.); elena.barbieri@uniurb.it (E.B.); 2 Chemistry Interdisciplinary Project (CHIP), School of Pharmacy, University of Camerino, 62032 Camerino, Italy; giovanni.caprioli@unicam.it (G.C.); agnese.santanatoglia@unicam.it (A.S.); sauro.vittori@unicam.it (S.V.)

**Keywords:** albedo, flavedo, bioactive compound, functional food

## Abstract

*Limoncella* of Mattinata, a rare and ancient Mediterranean citrus fruit, was investigated by sequence analysis of the ribosomal internal transcribed spacer regions, which assigns it as a variety of *Citrus medica* L. Morphological, chemical, and biomolecular approaches, including light and electron microscopy, HPLC-ESI-MS/MS, and antioxidant and anti-inflammatory assays, were used to characterize the flavedo and albedo parts, usually rich in bioactive compounds. The morphological findings showed albedo and flavedo cellular structures as “reservoirs” of nutritional components. Both albedo and flavedo hydroalcoholic extracts were rich in polyphenols, but they were different in compounds and quantity. The flavedo is rich in *p-coumaric* acid and rutin, whereas the albedo contains high levels of hesperidin and quercitrin. Antioxidant, anti-inflammatory, and genoprotective effects for albedo and flavedo were found. The results confirmed the health properties of flavedo and highlighted that albedo is also a rich source of antioxidants. Moreover, this study valorizes *Limoncella* of Mattinata’s nutritional properties, cueing its crops’ repopulation.

## 1. Introduction

*Limoncella* is the popular name of a fruit tree variety of the Rutaceae family, and it can be found in Mattinata, a location on the Southern Italian coast of the Gargano in the Puglia Region. This variety belongs to the *Citrus* genus and is a rare and ancient Mediterranean citrus fruit that is under-investigated compared to other citrus cultivars. We are convinced that *Limoncella* has nutritional and therapeutic properties, all to be discovered.

The Rutaceae family comprises 150 genera with approximately 2000 species, 70 of which are *Citrus*. The taxonomy of *Citrus* still stirs up severe doubts in the scientific community because of the intergeneric sexual compatibility, the high frequency of bud mutations, and the long history of cultivation and spread [1]. Despite the difficulties in establishing a consensual classification of edible citrus, most authors now agree on the origin of cultivated forms. The use of molecular markers and genome sequencing have contributed to identifying four primary taxa: *C. maxima* (pummelos), *C. medica* (citrons), *C. reticulata* (mandarins), and *C. micrantha* (a wild Papeda species) [2].

Citrus fruit peel (epicarp) is composed of flavedo (exocarp), the pigmented more external region, and albedo (mesocarp), the white layer before the pulp. This last part is more abundant in *Limoncella* than peel and is edible. Citrus fruits have a long history of use in pharmacology due to their rich content of bioactive compounds, particularly flavonoids, essential oils, and vitamin C. These components offer a range of therapeutic benefits and are utilized in various medicinal and health applications. Some species of *Citrus* have a broad spectrum of biological activities, including antibacterial, antiviral, antioxidant, antifungal, analgesic, and anti-inflammatory [3,4,5,6]. Many scientific studies refer to the citrus peel as a whole but must distinguish between the flavedo and albedo layers separately. The peel is often treated as a single entity in research, which can sometimes overlook the unique properties and functions of these individual components. Studies conducted on various Citrus species have shown that peels have antioxidant properties, but little is known about the albedo fraction [7,8,9,10,11,12]. The available literature data, however, reveal that albedo is characterized by high nutritional value due to the presence of functional compounds, such as phenolic acid, flavanones, and flavones [13].

The present work aimed to demonstrate the hypothesis that *Limoncella* is a precious, healthy fruit due to the properties of the flavedo and the albedo, this last section representing a substantial part of the fruit that the inhabitants of Mattinata willingly consume.

Having no knowledge of this variety, we performed preliminary studies, including sequence analysis of the ribosomal internal transcribed spacer regions to assign it a species of *Citrus*, as well as morphological characterization of flavedo and albedo to provide this kind of knowledge for the first time. Our main aim was to investigate properties, including antioxidant, anti-inflammatory, and genoprotective activities, to valorize its flavedo and albedo as a natural resource, and to promote the cultivation of this traditional citrus tree on the verge of extinction.

## 2. Materials and Methods

### 2.1. Materials

The fresh fruits of *Limoncella* were harvested in October 2018 and 2023 on a farm located in Mattinata (FG, Italy) and immediately processed for different purposes.

Cyanidin-3-glucoside chloride, delphinidin-3,5-diglucoside chloride, delphinidin-3-galactoside chloride, petunidin-3-glucoside chloride, malvidin-3-galactoside chloride, quercetin-3-glucoside, and kaempferol-3-glucoside were purchased from PhytoLab (Vestenbergsgreuth, Germany). The remaining 31 analytical standards of the 38 phenolic compounds were supplied by Sigma-Aldrich (Milan, Italy). Formic acid (99%) was obtained from Merck (Darmstadt, Germany). Analytical-grade hydrochloric acid (37%) was obtained from Carlo Erba Reagents (Milan, Italy). HPLC-grade methanol was supplied by Sigma-Aldrich (Milano, Italy). Deionized water (>18 MΩ cm resistivity) was further purified using a Milli-Q SP Reagent Water System (Millipore, Bedford, MA, USA). All solvents and solutions were filtered through a 0.2 μm polyamide filter from Sartorius Stedim (Goettingen, Germany). Before HPLC analysis, all samples were dissolved in methanol and filtered with a Phenex™ RC 4 mm 0.2 μm syringeless filter, Phenomenex (Castel Maggiore, BO, Italy). Reagents for sample preparation, antioxidant activity experiments, and genoprotective properties were purchased from Sigma Aldrich. Cell culture materials and reagents were from VWR International (Milan, Italy).

### 2.2. PCR Amplification of ITS Region

The molecular characterization was performed by amplification and sequencing of the ITS (internal transcribed spacer) region. According to the manufacturer’s recommendation, the genomic DNA was obtained from 100 mg plant leaf using a QIAamp DNA mini kit (Qiagen, Milan, Italy). Universal primers ITS1F-(5′-TCCGTAGGTGAACCTGCGG-3′) and ITS4 R-(5′-TCCTCCGCTTATTGATATGC-3′), located at 3′ and 5′ of the 18 S and 28 S of ribosomal genes, respectively, were employed. PCR reactions were performed in a 25 μL volume containing 50 ng of the genomic DNA, 0.4 μM of each of the above primers, 200 μM dNTP’s, 2.5 μL of 10× buffer, and 0.74 U of Taq DNA polymerase (Takara). Reaction conditions were as follows: denaturation at 95 °C for 10 min followed by 35 cycles of 95 °C for 30 s, 55 °C for 30 s, and 72 °C for 30 s, with final extension at 72 °C for 10 min. The amplification products were electrophoresed on 0.8% agarose TBE gel and stained with ethidium bromide (0.3 μg/mL). PCR products were purified using a gel extraction kit (Qiagen, Milano, Italy). Both strands of the products were sequenced using the pair of primers used in the PCR amplification. Sequences were run on an ABI 3700 automated sequencer, and the obtained sequences were aligned using BLAST as described in the Supplementary Material.

### 2.3. Light and Transmission Electron Microscopy

For light and transmission electron microscopy (TEM), the samples were cut into small pieces, fixed in 2.5% glutaraldehyde, and post-fixed in 1% aqueous osmium tetroxide, both in phosphate buffer for one hour. The samples were rinsed in phosphate buffer, dehydrated in an increasing ethanol series (50, 70, 80, 90, 95, 100%, 15 min each), further dehydrated twice with propylene oxide for 15 min, and then incubated in epoxy resin at 60 °C for three days. Semi-thin sections were cut with an ultra-microtome (Ultratome LKB) into 1 mm semi-thin sections and stained with 1% Toluidine blue in distilled water, 0.2% Basic fuchsin in ethanol, Safranin O in 50% alcohol, and 0.13% toluidine blue + 0.02% Azur II. Basic fuchsin, Safranin O, and Azur II staining were performed on etched resin sections with sodium meta-periodate. Different dyes were used to highlight the various parenchyma morphological components. From the same sample block, sections were cut into 70–80 nm ultra-thin sections and placed on 400 mesh grids. Ultra-thin sections were stained for 45 min each with Uranyless followed by 30 min lead citrate and observed by Philips CM10 at 80 kV.

### 2.4. Scanning Electron Microscopy (SEM) and Environmental Scanning Electron Microscopy (ESEM)

Sample preparation for conventional SEM analysis involved fixation in 2.5% glutaraldehyde (Sigma-Aldrich) for 1 h at 4 °C and dehydration using 50%, 70%, 80%, 90%, and twice with 100% ethanol (Sigma-Aldrich) for 5 min at each concentration. The samples were treated with hexamethyldisilazane (HMDS), transferred on aluminum stubs, and sputter-coated with an S-thin layer of gold, approximately 10 nm. Images were acquired using a Philips 515. ESEM technology represents an upgrade of the conventional scanning electron microscope (SEM), allowing the observation of samples at different vacuum levels, either prepared according to the conventional SEM method or natural, without dehydration or conductive coating. The latter opportunity is advantageous for biological samples, allowing morphological analysis without pretreatment before observation. If equipped with a spectrometer for the dispersion of energy (EDS), ESEM allows the semiquantitative detection of chemical elements constituting the ultrastructural components of the sample through point or areal analysis. An FEI Quanta 200 FEG Environmental Scanning Electron Microscope (FEI, Hillsboro, OR, USA) was used with an energy-dispersive X-ray spectrometer (EDAX Inc., Mahwah, NJ, USA). The analyses used a focalized electron beam in a vacuum electron gun pressure of 5.0 × 10^−6^ bar. The ESEM was utilized in low vacuum mode, with a specimen chamber pressure set at about 0.80 mbar, an accelerating voltage of 25 kV, a working distance of 10 mm, a tilt angle of 0, a variable beam diameter, and a magnification between 100 and 40,000×. The images were obtained utilizing the back-scattered electron detector to highlight the presence of particles or aggregates or the secondary electron detector to highlight the morphological features. The spectrometric unit has an ECON (Edax Carbon Oxygen Nitrogen) 6 UTW X-ray detector and Genesis Analysis software. Each sample was analyzed with a time count of 100 s and an Amp Time of 51, while the probe current was 290 μA.

### 2.5. Preparation of Albedo and Flavedo Extracts

Albedo and flavedo of Limoncella lemons from Mattinata were separated and subject to drying at 60 °C overnight on air in the stove. After drying, the albedo and peel were triturated in a mortar with liquid nitrogen. This procedure was repeated three times to reduce the samples to a fine powder. Subsequently, 3 g of ground flavedo and albedo samples were extracted with 30 mL of 80% ethanol/water (80:20 *v*/*v*) at 4 °C under stirring. The albedo and flavedo extracts were then centrifuged at 400× *g* for 15 min, and the residues were extracted using two additional portions of the ethanol/water mixture. All supernatants were pooled, concentrated, and kept at −20 °C until use.

### 2.6. Quantification of Total Phenolics by Folin–Ciocalteu Method

Total polyphenol content was determined using the Folin–Ciocalteu method described by Singleton et al. [14]. The total volume of the reaction mixture was minimized to 1 mL. The extract solutions (10 μL) were mixed with Folin–Ciocalteu reagent (50 μL) and deionized water (90 μL). Three minutes later, 300 μL of 20% (*w*/*v*) sodium carbonate was added, and the mixture was brought up to 1 mL with distilled water. The tubes were vortex-mixed for 15 s and allowed to stand for 30 min at room temperature for color development. Absorbance was then measured at 725 nm using a UV Beckman spectrophotometer. The amount of total phenolics was expressed as caffeic acid equivalents through the calibration curve of caffeic acid. The calibration curve ranged from 1 to 15 μg/mL (R^2^ = 0.9973).

### 2.7. HPLC-ESI-MS/MS Analysis

HPLC-MS/MS studies were performed using an Agilent 1290 Infinity series and a Triple Quadrupole 6420 from Agilent Technology (Santa Clara, CA, USA) equipped with an electrospray ionization (ESI) source operating in negative and positive ionization modes by following a previously published method [15,16,17]. The separation of target compounds was achieved on a Synergy Polar–RP C18 analytical column (250 mm × 4.6 mm, 4 µm) from Phenomenex (Chesire, UK). The column was preceded by a Polar RP security guard cartridge (4 mm × 3 mm ID). The mobile phase was a mixture of water (A) and methanol (B), with formic acid 0.1% at a flow rate of 0.8 mL min^−1^ in gradient elution mode. The composition of the mobile phase varied as follows: 0–1 min, isocratic condition, 20% B; 1–25 min, 85% B; 25–26 min, isocratic condition, 85% B; 26–32 min, 20% B. The injection volume was 2 μL. The column temperature was 30 °C, and the drying gas (N_2_) temperature in the ionization source was 350 °C. The gas (N_2_) flow was 12 L/min, the nebulizer pressure was 55 psi, the capillary voltage was 4000 V, and a gas flow rate was maintained at 12,000 mL/min. Detection was performed in the “dynamic-multiple reaction monitoring” (dynamic-MRM) mode, and the dynamic-MRM peak areas were integrated for quantification. The most abundant product ion was used for quantitation, and the other for quantification. The selected ion transitions and the mass spectrometer parameters, including the specific time window for each compound (Δ retention time), are reported in Table S2 (Supplementary Materials).

### 2.8. Antioxidant Activity

#### 2.8.1. DPPH (2,2-Diphenyl-1-picrylhydrazyl) Assay

The DPPH radical scavenging assay was conducted using the procedure previously described by Saltarelli et al. [18]. Fresh DPPH ethanol solution (850 µL, 100 μM) was mixed with the sample at different concentrations (150 µL, 0.2–3.5 mg/mL). The decreased absorbance at 517 nm was recorded after 10 min at room temperature. The scavenging activity was calculated as % = [(A_0_ − A)/A_0_] × 100, where A_0_ is the absorbance of the control reaction, and A is the absorbance in the presence of samples. The EC_50_ value was calculated from the plots as concentration extracts required to provide 50% free radical scavenging activity.

#### 2.8.2. ABTS, 2,2,-azino-*bis*(3-Ethylbenzothiazoline-6-sulphonic acid) Assay

Antioxidant activity against ABTS radical was performed as described by Loizzo et al. with some modifications [19]. Briefly, the reaction mixture was prepared by mixing 7 mM ABTS solution and 2.45 mM potassium persulphate followed by 12–16 h incubation in the dark at room temperature to produce ABTS radical. Before use, the solution was diluted with ethanol to absorb 0.80 ± 0.05 at 734 nm. Aliquots of the sample at concentrations ranging from 0.01 to 0.4 mg/mL were added to 1 mL of ABTS ethanolic solution and incubated in the dark at room temperature for 6 min. The absorbance was then recorded at 734 nm using a UV Beckman spectrophotometer. The ABTS radical scavenging activity was calculated following the equation: ABTS scavenging activity (%) = [(A_734_ nm of blank − A_734_ nm of the sample)/A_734_ nm of blank] × 100. Results are reported as EC_50_ values (µg/mL). Trolox was a reference compound (0.5–5 μg/mL).

#### 2.8.3. Lipoxygenase Inhibition Assay

Soybean 5-lipoxygenase was used for the assay, according to Saltarelli et al. [18]. Inhibition experiments were performed by measuring the loss of 5-lipoxygenase activity (0.18 µg/mL) with 100 µM linoleic acid as the substrate in 50 mM sodium phosphate, pH = 6.8. The reaction mixture was pre-equilibrated at 20 °C for 20 min without the enzyme. Inhibition studies in the presence of various extract concentrations (0.2–0.7 mg/mL) were recorded at 235 nm at 20 °C using a UV Beckman spectrophotometer. The lipoxygenase activity was calculated as % = 100 − {[(Δ_235_ nm of blank − Δ_235_ nm of sample)/Δ_235_ nm of blank] × 100}. EC_50_ was determined by plotting the graph with the concentration of extracts versus the percentage of inhibition of linoleic acid peroxidation.

#### 2.8.4. Chelating Capacity on Fe^2+^

Fe^2+^ chelating capacity was evaluated as described by Saltarelli et al. [18] with some modifications. Briefly, an aliquot of 20 μL of FeSO_4_ solution (2 mM) was added to 200 μL of sample (range 20–125 μg/mL) and incubated at room temperature for 5 min. After that, an aliquot of 40 μL of ferrozine solution (4 mM) was added to the reaction mixture, and the sample volume was diluted to 1 mL with deionized water, mixed, and incubated for 10 min in the dark at room temperature. The absorbance at 562 nm was then spectrophotometrically determined. The chelating activity was calculated as % = [(A562 nm of blank − A562 nm of the sample)/A_562_ nm of blank] × 100. EC_50_ is the concentration at which ferrous ions are chelated by 50%, and this was evaluated by plotting the sample concentration versus the chelating activity.

### 2.9. DNA Nicking Assay

The DNA nicking assay evaluated the protective effect of hydroalcoholic albedo and Limoncella flavedo extracts against oxidative DNA damage, which employs ferrous ions and dioxygen (Fe^2+^ + O_2_) to generate DNA strand breaks induced from free radicals [20]. The assay consists of a cell-free system composed of plasmid DNA (pEMBL8), which resembles the structure of mtDNA [21]. The hydroalcoholic solutions were sequentially diluted with water to obtain the final concentrations of 250 µg/mL, 125 µg/mL, 63 µg/mL, and 31 µg/mL. Each extract was assayed in a final volume of 72 µL, consisting of PBS (phosphate buffer solution), 7 µg/mL of pEMBL8, and 20 µL of the related dilution. The mixtures were added 8 μL of 3 mM FeSO_4_, freshly prepared and kept on ice. The tubes were incubated for 10 min at 37 °C, and the reaction was stopped with 20 µL of loading buffer. The disappearance of the supercoiled form of the plasmid (CCC) was assessed with ethidium bromide-stained agarose gel electrophoresis followed by quantification using Gel Doc 2000 and Quantity One software (Bio-Rad, Milan, Italy). In detail, 20 µL of each sample were loaded onto a 1.2% *w/v* agarose gel in TAE buffer (40 mM Tris-acetate and 1 mM EDTA, pH 8.0) and run at 80 V for 30 min in a small electrophoresis chamber (Mini-Sub Cell GT Systems, Bio-Rad). The gel was stained with 0.3 μg/mL ethidium bromide (EtBr). The EC_50_ value was calculated by determining the concentration of the compound protecting half of the supercoiled plasmid.

### 2.10. Cell Cultures

The human keratinocyte cell line HaCaT was obtained from the Interlab Cell Line Collection (ICLC, Genoa, Italy). Cells were grown in DMEM medium supplemented with 10% fetal bovine serum, 2% glutamine, 1% sodium pyruvate, and 100 U/mL penicillin/streptomycin. Cells were maintained in an incubator at 37 °C and 5% CO_2_. RAW 264.7 murine macrophages were cultured in RPMI 1640 medium with 10%, 2 mM glutamine, and 1% 100 U/mL penicillin/streptomycin, and maintained at 37 °C in a 5% CO_2_ atmosphere.

### 2.11. Cytotoxicity Assays

The cytotoxic effects of the Limoncella hydroalcoholic albedo and flavedo extract against HaCaT cells were analyzed by WST-8 and sulforhodamine B (SRB) assays, which evaluate cellular metabolic activity and cellular protein content, respectively [22]. In detail, cells (5 × 10^3^/well) were seeded in 96-well plates and treated with water-diluted extracts from 110 to 7 µg/mL. After 24 h of incubation, the test compounds were removed, and a fresh medium containing WST-8 (Sigma-Aldrich, Milan, Italy) was added to each well. Cells were further incubated at 37 °C for up to 4 h, and color development was monitored at 450 nm in a microplate reader (Multiskan FC, Thermo Scientific, Dreieich, Germany) [23]. As previously published, the SRB assay was also performed in the same 96-well plate [24]. Briefly, cells were fixed with cold 50% trichloroacetic acid and stained with 0.4% SRB (Sigma-Aldrich, Milan, Italy) dissolved in 1% acetic acid. The protein-bound dye was subsequently solubilized with 10 mM Tris, and the absorbance was read at 570 nm in a microplate reader (Multiskan FC, Thermo Scientific). The concentration that caused 50% growth inhibition (IC_50_) was calculated, and the data were expressed as a percentage (%) compared to that of untreated cells (controls).

### 2.12. Evaluation of Antioxidant Properties by DCFH-DA Assay

The antioxidant properties of albedo and flavedo extracts were analyzed in HaCaT cells using 2′,7′-dichlorofluorescein diacetate (DCFH-DA, Sigma-Aldrich, Milan, Italy), which transforms into 2′,7′-dichlorofluorescein (DCF) and is highly fluorescent after oxidation [23]. In detail, cells (1 × 10^4^/well) were seeded into black 96-well plates and incubated for 2 h with a concentration of 25 µg/mL extracts in 100 µL DMEM. The medium was then removed and replaced with 50 µL DCFH-DA (5 µM in PBS), incubated for 30 min at 37 °C. After the removal of the excess probe, cells were treated with 100 µL of hydrogen peroxide (H_2_O_2_, 100 μM in PBS) for 30 min, and DCF fluorescence emission was measured at ex/em 485/520 nm in the FluoStar multi-well plate reader Optima (BMG Labtech, Germany). Data were expressed as relative oxidation compared to non-oxidized cells.

### 2.13. Determination of Nitric Oxide Production

The anti-inflammatory properties of both albedo and flavedo hydroalcoholic extracts were evaluated in RAW 264.7 cells (murine macrophages) stimulated by lipopolysaccharide (LPS) (Sigma-Aldrich, Milan, Italy). Cells (3 × 10^4^/well) were seeded into 96-well plates and treated with both Limoncella extracts (50 µg/mL) in the presence and absence of 1 µg/mL LPS for 24 h. The cells were also incubated alone in the presence and absence of LPS as a control. The drug dexamethasone at a 5 µg/mL concentration was used to validate the test. Subsequently, nitric oxide (NO) levels were determined in the supernatant medium using Griess reagent (Sigma-Aldrich, Milan, Italy) [25]. Absorbance was measured at 570 nm using a plate reader (Bio-Rad Laboratories, Milan, Italy).

### 2.14. Statistical Analysis

Statistical analysis was performed using the computer program Graph Pad Prism (v4.0, GraphPad Software Inc., San Diego, CA, USA). Data were expressed as mean values ± standard deviation (SD) of three independent experiments (*n* = 3) and analyzed by one-way analysis of variance (ANOVA) followed by Dunnett’s or Tukey’s test. *p*-values less than 0.05 were referred to as significantly different between variables.

## 3. Results

### 3.1. PCR Amplification, DNA Sequencing, and Sequence Analysis of the ITS Region

The ribosomal DNA region, including the ITS1, 5.8S, and ITS2, was amplified by polymerase chain reaction using universal primers located on the 3′ end of 18S and the 5′ start of 28S. The electrophoretic patterns showed a ~700 bp amplicon PCR product, purified and sequenced. Sequence alignment analyses assign this fruit to *Citrus medica* species (Table S1 and Figure S1 in Supplementary Material).

### 3.2. Morphological and Semi-Quantitative Data

**Figure 1:**
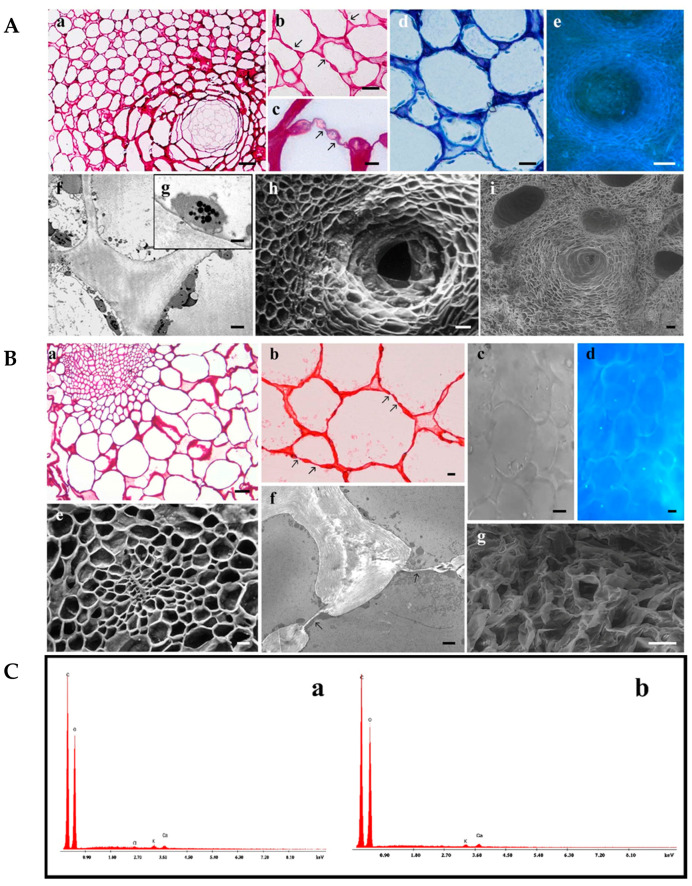
Morphological analysis and semi-quantitative data of flavedo and albedo from Limoncella. (**A**) Flavedo parenchymal cells were observed by LM (a–d), fluorescent (e), TEM (f,g), SEM (h), and ESEM (i) microscopy. Basic fuchsin (a–c) staining highlighted cellular wall and plasmodesmata (arrows). Toluidine Blue (b) evidenced cytoplasmic organelles and central vacuoles. In (f,g), some plastids are visible. (a,h,i) indicate the oil gland morphology. (a,e) Bar = 5 μm; (b,f) Bar = 2.5 μm; (d) Bar = 1 μm; (g) Bar = 0.5 mm; (h) Bar = 50 μm; (i) Bar = 100 μm.pp. (**B**) Albedo parenchymal cells were observed by LM and arrows indicate plasmodesmata (a,b), differential interference contrast (c), fluorescent (d), SEM (e), TEM (f), and ESEM (g) microscopy. Basic fuchsin (a) and safranin (b) staining are used to identify cellular walls. In (f), plasmodesmata structures were visible. (a,b,e,g) Bar = 20 μm; (c,d) bar = 0.2 μm; (f) bar = 2 μm. (**C**): Semi-quantitative analysis performed on the albedo (a) and flavedo (b) surfaces.

All morphological approaches allowed us to observe that the flavedo (Figure 1A) consist of undifferentiated tightly packed parenchyma tissue with slightly turgid cells, which present numerous large oil glands (a, e, h, i), formed inside by cells with very thin walls, as demonstrated in A. Some plastids (f, g) and plasmodesmata structures (b, c) can be observed. The albedo parenchymal cells (Figure 1B) show a thin wall and a considerable central vacuole. Plasmodesmata or intercellular bridges that connect plant cells are cylindrical channels approximately 40 nm in diameter, which are characteristic structures that enhance cell communication through the secondary wall (b, f). Semi-quantitative chemical analyses showed that both the albedo (a) and flavedo (b) contained carbon (a 73.79%; b 63.75%), oxygen (a 25.47%; b 35.96%), chloride (a 0.16%), potassium (a 0.27%; b 0.34%), and calcium (a 0.31%; b 0.50%) (Figure 1C).

### 3.3. Characterization and Quantification of Phenolic Compounds

The Folin–Ciocalteu method (FC) and HPLC-ESI-MS/MS were used to evaluate the polyphenol content. Both methods showed that the total phenol content was comparable in both extracts (Table 1). In particular, the total phenol content measured by FC was 18,700 ± 323 and 22,040 ± 853 mg/kg dw in albedo and flavedo extracts, respectively.

In contrast, the values obtained by HPLC were slightly lower than those observed by FC (14,863.69 and 11,392.24 mg/kg dw for albedo and flavedo extracts, respectively). The phenolic compounds were characterized by HPLC-ESI-MS/MS testing the presence of 38 compounds, as reported in Table 1. It is worth underlining the higher concentration of hesperidin (15-fold) and quercitrin (7-fold) in the albedo compared to flavedo.

### 3.4. Antioxidant Activities

**Figure 2:**
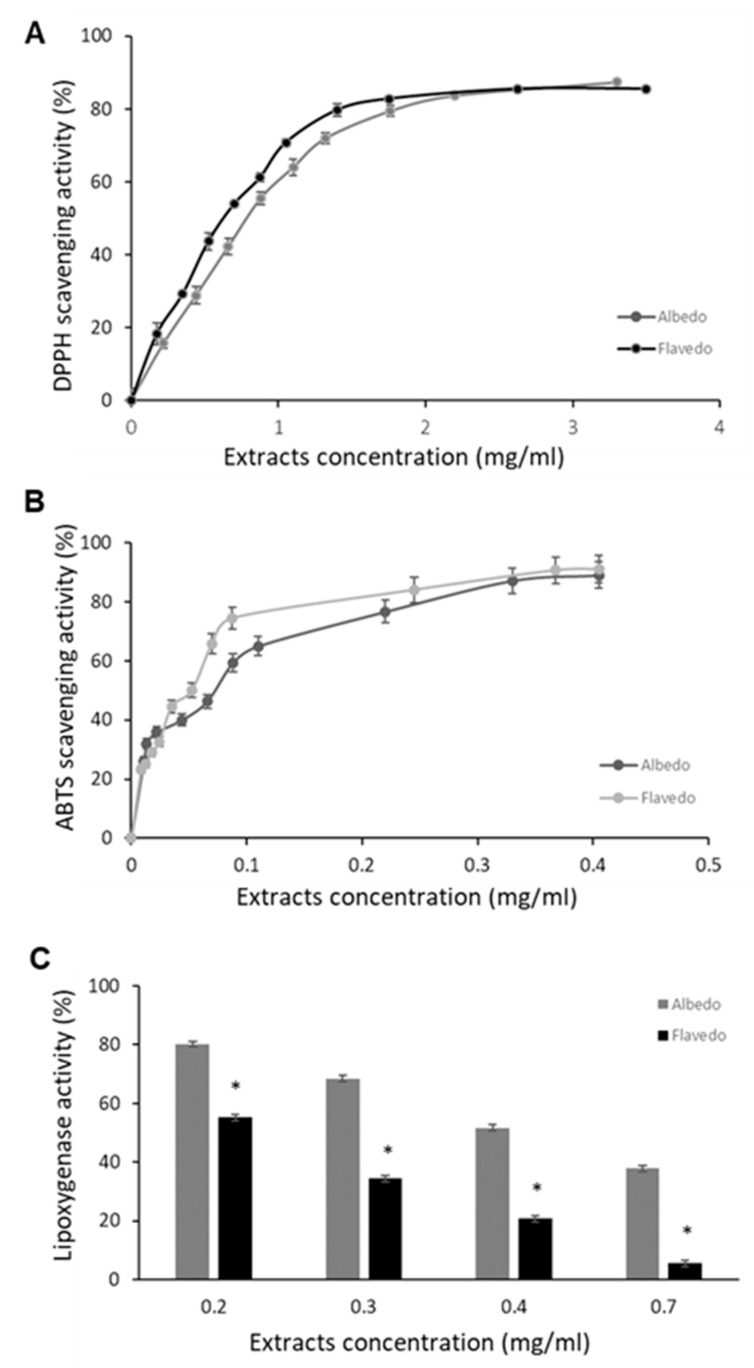
Antioxidant capacity of albedo and flavedo *Limoncella* extracts. Scavenging effect on DPPH (**A**) and ABTS (**B**) tests. Effect on lipoxygenase activity in vitro (**C**). The data represent the inhibition percentage induced by increasing albedo and flavedo extracts. Data are expressed as mean ± SD (n = 3). * *p* < 0.05.

The antioxidant capacity of albedo and flavedo extracts was evaluated using different chemical tests, namely the DPPH assay, the ABTS assay, the chelating ability of Fe^2+^, and the lipoxygenase inhibition assay. Figure 2 shows the antioxidant activity of the albedo and flavedo extracts from *Limoncella.* The free-radical scavenging effect of *Limoncella* extracts was evaluated using DPPH and ABTS assays. As reported in Figure 2A,B, albedo and flavedo extracts showed scavenging activity in a dose-dependent manner with an inhibition range of 80–85% at a concentration >0.2 and 2.0 mg/mL in ABTS and DPPH tests, respectively. Both parts of the fruit prevented oxidative damage mediated by free radicals since there was no statistically significant difference between albedo and flavedo extracts. The albedo extract did not show the chelating effect on Fe^2+^, and flavedo displayed only a small chelating activity: about 20% at 15 mg/mL. Furthermore, the *Limoncella* extracts inhibited the oxidation of linoleic acid catalyzed by lipoxygenase in a dose-dependent manner (Figure 2C). However, the flavedo extract was significantly more effective than albedo, since at 0.7 mg/mL of extract concentration, lipoxygenase activity was 5.5 ± 2.5 and 37.7 ± 6.2, respectively.

EC_50_ values (Table 2) for ABTS and DPPH scavenging activity were comparable in the albedo and flavedo extracts. However, ABTS EC50 measurements were lower than the ones obtained for DPPH EC_50_ (about 0.063 mg/mL and 0.971–0.996 mg/mL, respectively). The antioxidant capacities of albedo and flavedo extracts were 35.42 ± 3.31 and 40.41 ± 10.15 µg Trolox equivalent/mg, respectively. A different EC_50_ level between albedo and flavedo extracts was detected for lipoxygenase inhibition; in particular, the EC_50_ of flavedo was significantly lower than that reported for albedo (Table 2).

### 3.5. Genoprotective Activity

**Figure 3:**
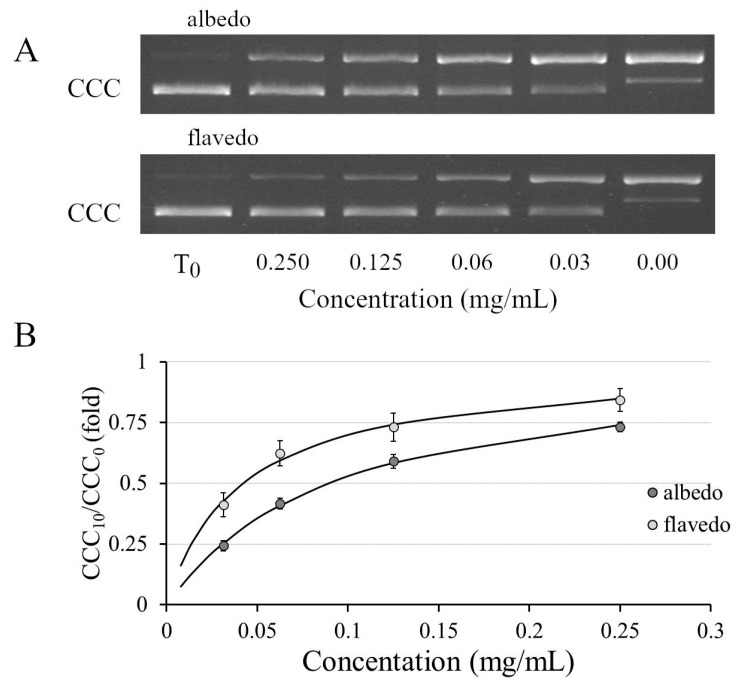
Protective activity of albedo and flavedo extract versus oxidative DNA damage evaluated by DNA nicking assay. (**A**) reports a representative agarose gel electrophoresis of the pEMBL8 samples before (T0) and after treatment with different concentrations of hydroalcoholic extracts. CCC is a supercoiled form of a plasmid. (**B**) reports the quantification analyses expressed as the ratio between the CCC after treatment and CCC at time 0.

DNA nicking assay is employed to check the ability of albedo and flavedo extracts against the plasmid DNA damage caused by free radicals generated in vitro by Fe^2+^ + O_2_. Figure 3A depicts the ability of the extracts to protect supercoiled DNA form (CCC) during the incubation with 3 mM Fe^2+,^ and Figure 3B reports the quantification analyses expressed as the ratio between the CCC after treatment and CCC at time 0. From Figure 3A, the protection of the supercoiled form of DNA is observed as the concentrations of the two extracts increase in the presence of the oxidant. This protection is higher with flavedo extracts compared to albedo. The bands of the open circular form (OC), formed in damaged DNA, are more evident with the albedo extract. This indicates that flavedo offers more excellent protection against DNA oxidation. The bands were quantified, and the densitometric analysis showed a dose-dependent response with a higher percentage of protection in flavedo (Figure 3B). The EC_50_ values obtained in the DNA nicking assays were 0.090 mg/mL and 0.042 mg/mL for albedo and flavedo, respectively.

### 3.6. Evaluation of the Cytotoxic Effects

**Figure 4:**
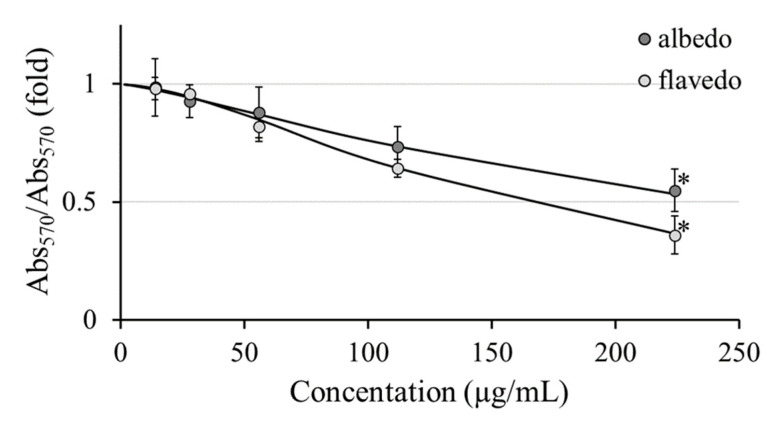
Cell viability evaluation by SRB colorimetric assay upon *Limoncella* extracts administered (from 14 to 224 µg/mL) to HaCaT cell lines for 24 h. The cell viability was expressed as the ratio between the absorbance at 570 nm of treated and untreated samples. Data are expressed as the mean ± SD (*n* = 3). * *p* < 0.05 vs. Ctrl.

The evaluation of the cytotoxicity of the hydroalcoholic extracts of *Limoncella* using the sulforhodamine B (SRB) test revealed a significant reduction in the viability of cultured human keratinocytes cells at concentrations higher than 200 µg/mL (Figure 4). The calculated IC_50_ values were 250 µg/mL and 161 µg/mL for albedo and flavedo, respectively.

### 3.7. Radical Scavenging Properties of Limoncella Extracts by Cell-Based Assay

**Figure 5:**
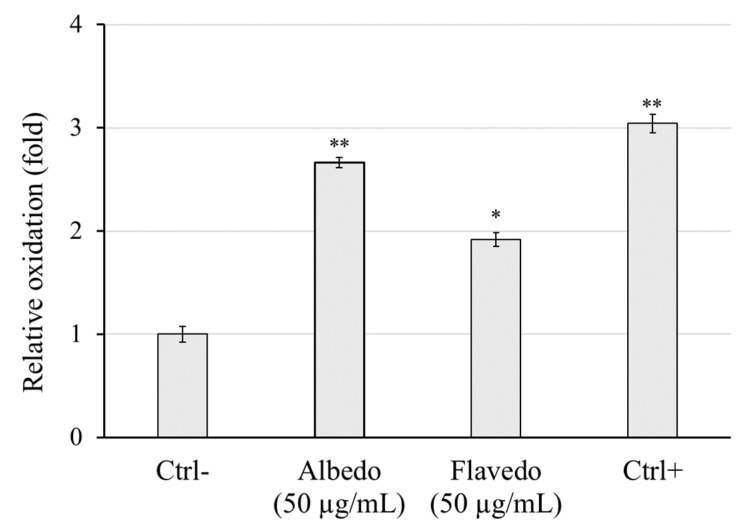
Evaluation of the antioxidant properties of albedo and flavedo extract in HaCaT cells. The relative intracellular oxidation levels were obtained by incubating the cells with 50 µg/mL of each extract. The untreated and non-oxidized cells were reported as Ctrl− while the untreated and oxidized (H_2_O_2_) cells were Ctrl+. Data are expressed as mean ± SD (*n* = 3). * *p* < 0.05 vs. Ctrl−. ** *p* < 0.01 vs. Ctrl−.

Radical scavenging properties of *Limoncella* extracts were also tested in a cell system. As reported in Figure 5, the administration of H_2_O_2_ to HaCaT cells (Ctrl+) led to a significant increase in DCF fluorescence emission compared to untreated cells (Ctrl−). When cells were pretreated for 2 h with 50 µg/mL of both *Limoncella* preparations, a highly significant reduction in H_2_O_2_-induced free radicals in flavedo extract was observed (−37-fold vs. Ctrl+), while a reduction less significant was observed in albedo extract (−12.5-fold vs. Ctrl+), further supporting their action as a protective antioxidant against oxidative stress.

### 3.8. Anti-Inflammatory Activity

**Figure 6:**
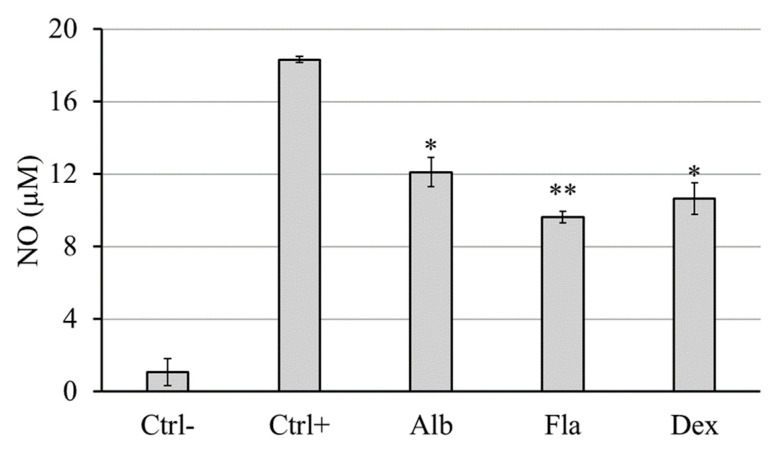
Extracellular NO release after RAW 264.7 stimulation by LPS for 24 h in the presence of albedo and flavedo extracts (50 µg/mL). Ctrl−: cell without stimulus or extract; Ctrl+: cell stimulated by 1 µg/mL LPS; Alb: 50 µg/mL albedo + 1 µg/mL LPS; Fla: 50 µg/mL flavedo + 1 µg/mL LPS; Dex: 5 µg/mL dexamethasone + 1 µg/mL LPS. Data are expressed as the mean ± SD (*n* = 3). * *p* < 0.05, ** *p* < 0.01 vs. Ctrl+ (Tukey’s post hoc test).

As reported in Figure 6, the stimulation of RAW 264.7 cells (murine macrophages) by 1 µg/mL lipopolysaccharide (Ctrl+) led to a robust extracellular release of the inflammatory response mediator NO as compared to unstimulated control cells (Ctrl−). When LPS-exposed cells were co-incubated with 50 µg/mL of both extracts, a decrease in NO production was observed; albedo extract reduced the production of NO at 12.1 µM and flavedo at 9.6 µM, respectively, compared to a positive control at 18.3 µM. The anti-inflammatory activity of both flavedo and albedo extract was comparable to that of the reference drug dexamethasone 5 µg/mL (10.6 µM vs. Ctrl+).

## 4. Discussion

The various species of *Citrus* are mainly used in the food and cosmetic fields. However, their antioxidant, antimicrobial, and anticancer properties have also found applications in the therapeutic field [26,27,28,29]. Most studies have been performed on the flavedo or the entire peel, while little is known about the beneficial properties of the albedo [12,30,31,32]. The present work represents an attempt to investigate and compare this part of the fruit with flavedo in an unknown ancient Mediterranean citrus variety from Southern Italy, of which we also performed further characterization analyses, including phylogenetic and morphological ones. The morphological evidence obtained utilizing different and complementary approaches allowed us to better understand the albedo’s and flavedo’s components that constitute the “reservoir” of nutritional substances and the parts of them that do not interfere with the extractive procedure. Concerning the chemical characterization of flavedo and albedo alcoholic extracts, herein, we report that they are rich in polyphenols, quite different in compounds and quantity (Table 1), although their total polyphenol content falls within the range of those previously reported for 35 cultivars of *Citrus reticulata* Blanco [33] and *Citrus medica* L. [34]. The total polyphenol content values obtained by HPLC were 14.86 and 11.39 g/kg dw for albedo and flavedo extracts, respectively, and they were slightly lower than those observed by the Folin–Ciocalteu method (18.70 ± 0.32 and 22.04 ± 0.85 g/kg dw in albedo and flavedo extracts, respectively). This outcome could be explained considering that the FC method estimates the content of reducing compounds, which are both phenolic and non-phenolic. In contrast, the HPLC analysis only measures the content of the major phenolics in both extracts. Notably, the flavedo was found to be rich in *p-coumaric* acid (1.56 g/kg) and rutin (5.99 g/kg), while the albedo contained high levels of hesperidin (11.62 g/kg) and quercitrin (1.96 g/kg). These findings suggest that different parts of *Limoncella* fruit have distinct health-promoting properties that could be harnessed in food and therapeutic applications. Hesperidin is present in the highest amounts in *C. sinensis* (sweet orange), with a concentration of 28.6 mg/100 mL in the whole fruit. The albedo tissue contains the highest amount of hesperidin, while the juice vesicle tissue has the lowest. In contrast, a negligible amount of hesperidin is found in *C. aurantium* [35]. The qualitative and quantitative differences of the polyphenolic compounds also explain the different activities or functions of the phytocomplexes of these two parts of the fruit [36]. The qualitative/quantitative characterization of the two ethanolic extracts led us to test their antioxidant activity, which cell-free and cell-based models performed. DPPH and ABTS assays are cell-free methods showing that either flavedo or albedo exhibit similar radical scavenging properties. Furthermore, these values are comparable to those previously reported for *Citrus lumia* Risso [12]. In particular, results from the DPPH EC_50_ values showed the same order of magnitude of radical scavenging activity for both Italian species, being 0.996 ± 0.17 for *Limoncella* and about 0.533 for *Citrus lumia*. The flavedo extract is more performant in the assays investigating the effect of Fe^2+^ chelating, lipoxygenase activity, and the level of ROS.

We evaluated the antioxidant properties of two extracts using a cell-based method with the DCFA-DA probe in H_2_O_2_-treated HACAT cells. This test requires previous viability assays to select the range of possible non-cytotoxic concentrations and the toxicity threshold. Cell viability was assessed using the WST-8 and SRB assays. While the SRB assay gave significant results for both samples, the WST-8 assay gave false results [37]. The cytotoxicity evaluation against HaCaT cells indicated that both extracts are weakly cytotoxic at higher concentrations, with an IC_50_ value of 161 µg/mL for flavedo and 250 µg/mL for albedo. Importantly, these concentrations are significantly higher than the EC_50_ values obtained in the antioxidant and genoprotective assays, suggesting that the extracts can provide health benefits at non-toxic concentrations. [38]. Both *Limoncella* extracts significantly reduced H_2_O_2_-induced free radicals, with the flavedo extract showing a more pronounced effect than the albedo extract, reinforcing their role as protective antioxidants against oxidative stress. The lipoxygenase enzymes contribute to the emergence of inflammation and allergic reactions by producing leukotrienes. Soybean lipoxygenase has substrate selectivity and inhibitory properties similar to humans, exhibits good stability, and was used in inhibition assay [39]. Albedo and flavedo extracts significantly inhibited lipoxygenase action with an EC_50_ of 0.54 ± 0.10 0.14 ± 0.06 for albedo and flavedo, respectively. In the literature, it was reported that coumarin and hesperidin show considerable lipoxygenase inhibitory activity [39,40], and, as reported above, these phenolic compounds are present in large amounts in *Limoncella* extracts. The oxidation of linoleic acid catalyzed by lipoxygenase suggested the anti-inflammatory activity of the extracts, which was further confirmed using the Griess test with RAW 264.7 macrophages [41]. The flavedo showed greater anti-inflammatory activity than the albedo (−0.54-fold vs. −0.67-fold relative to Ctrl+), similar to the effect of dexamethasone (−0.60-fold). This anti-inflammatory activity is consistent with the known properties of polyphenols.

The anti-inflammatory properties of lemon peel extract may be attributed to *p-coumaric* acid, which is ten times more abundant in flavedo than albedo and has shown immunosuppressive effects by reducing immune responses and macrophage activity in rats [42]. Additionally, at 40-times higher concentrations in flavedo, rutin exhibits anti-inflammatory effects by inhibiting inflammatory mediators, which may help alleviate conditions like arthritis and prevent chronic diseases [43]. Other unidentified anti-inflammatory compounds may also be present in the extracts. Fruits and vegetables are the main anticancer foods, and they are rich in antioxidants, Vitamins C and E, beta-carotene, and lipothin. Citrus fruits, in particular, play an essential role as genoprotective agents. We evaluated the genoprotective effects of albedo and flavedo extracts using a DNAnicking assay. Both extracts showed significant antioxidant protection against hydroxyl radicals generated in vitro [44,45,46]. As previously discussed, the IC_50_ for cytotoxicity in HaCaT cells (albedo: 250 µg/mL; flavedo: 161 µg/mL) was much higher than the EC_50_ in the DNA assay (albedo: 90 µg/mL; flavedo: 42 µg/mL), indicating their safety at protective concentrations. Both extracts protected the plasmid DNA, which resembles the structure of mtDNA, from oxidative damage, suggesting potential benefits for mitochondria and protection against age-related oxidative damage [47].

Many studies have been performed on the genoprotective properties of lemon juice and peel. A study conducted on *Citrus medica* L. through the Ames test demonstrated the antimutagenic and anticarcinogenic effects of the juice [48]. Koolaji et al. found that citrus peel contains numerous bioactive compounds, including carotenes, essential oils, pectins, and phenolic compounds, which may reduce cancer risk [49]. The peel extract of the genus *Citrus* has a higher antitumor activity than the single isolated compound.

Our study primarily focuses on in vitro assays to assess the bioactive properties of *Limoncella* extracts. While the results indicate promising antioxidant and anti-inflammatory effects, further studies could be aimed at demonstrating these benefits and evaluating their efficacy and safety in more complex biological models. Additionally, the different environmental and growth conditions of *Limoncella* could significantly influence the polyphenolic content, which may affect the reproducibility and consistency of the bioactive effects observed.

The bioactive compounds identified in *Limoncella* flavedo and albedo, particularly hesperidin, quercitrin, and rutin, have been associated with various health benefits, including antioxidant and anti-inflammatory activities [43,50,51,52,53] These properties make *Limoncella* an attractive candidate for developing functional foods and nutraceuticals and suggest potential applications in the cosmetic industry.

## 5. Conclusions

In conclusion, this study demonstrates that *Limoncella* is a valuable source of bioactive compounds with numerous potential applications in food science, medicine, and cosmetics. Our findings indicate that both flavedo and albedo possess beneficial functional properties, with the albedo being particularly rich in antioxidants. These bioactive properties suggest that *Limoncella* could be integrated into functional products with significant health benefits, such as antioxidative, anti-inflammatory, and genoprotective effects.

As an ancient variety of *Citrus medica* L. that remains largely under-researched, *Limoncella* holds potential for both scientific exploration and practical application. This research aims to valorize *Limoncella* and promote its cultivation as a sustainable strategy, contributing to biodiversity conservation and personalized nutrition. Further studies could focus on refining cultivation practices, understanding bioactive efficacy in broader biological contexts, and integrating *Limoncella* extracts into disease prevention strategies. Promoting *Limoncella* crop repopulation and encouraging its consumption, including the albedo, could help preserve this ancient fruit and its beneficial properties.

## Figures and Tables

**Table 1:** Concentration (mg kg^−1^) of bioactive compounds in albedo and flavedo extracts (*n* = 3).

No	Compounds	Albedo Concentration mg kg^−1^	Flavedo Concentration mg kg^−1^
1	Gallic acid	5.57	41.20
2	Neochlorogenic acid	n.d. ^a^	n.d.
3	Delphinidin-3-galactoside	n.d.	7.35
4	(+)-Catechin	n.d.	n.d.
5	Procyanidin B2	n.d.	n.d.
6	Chlorogenic acid	0.80	1.54
7	p-Hydroxybenzoic acid	411.34	895.60
8	(-)-Epicatechin	n.d.	0.69
9	Cyanidin-3-glucoside	n.d.	n.d.
10	Petunidin-3-glucoside	0.45	1.82
11	3-Hydroxybenzoic acid	n.d.	n.d.
12	Caffeic acid	6.92	n.d.
13	Vanillic acid	134.39	n.d.
14	Resveratrol	n.d.	n.d.
15	Pelargonidin-3-glucoside	n.d.	0.50
16	Pelagonidin-3-rutinoside	n.d.	n.d.
17	Malvidin-3-galactoside	n.d.	n.d.
18	Syringic acid	n.d.	36.43
19	Procyanidin A2	n.d.	n.d.
20	p-Coumaric acid	143.89	1566.65
21	Ferulic acid	147.86	609.96
22	3,5-Dicaffeoylquinic acid	n.d.	n.d.
23	Rutin	137.04	5996.78
24	Hyperoside	n.d.	245.49
25	Isoquercitrin	9.73	216.05
26	Delphindin-3,5-diglucoside	68.98	212.24
27	Phloridzin	0.82	n.d.
28	Quercitrin	1961.17	267.76
29	Myricetin	0.41	n.d.
30	Naringin	3.86	1.65
31	Kaempferol-3-glucoside	47.45	4.73
32	Hesperidin	11,625.45	778.88
33	Ellagic acid	n.d.	24.49
34	trans-cinnamic acid	13.23	4.09
35	Quercetin	12.92	73.08
36	Phloretin	n.d.	n.d.
37	Kaempferol	128.20	246.73
38	Isorhamnetin	3.22	158.56
Total content (mg kg^−1^)		14,863.69	11,392.24
Total content (%)		1.48	1.14
Total content (mg kg^−1^) Folin–Ciocalteu method		18,700	22,040

^a^ n.d., not detectable. Relative standard deviation (RSD) for all compounds ranged from 2.54 to 10.31%.

**Table 2:** Antioxidant activities of albedo and flavedo extracts.

Part of Fruit	DPPH EC_50_ (mg/mL)	ABTS EC_50_ (mg/mL)	Lipoxygenase EC_50_ (mg/mL)	Total Antioxidant Capacity (µg TE/mg)
Albedo	0.996 ± 0.17	0.063 ± 0.029	0.54 ± 0.10	35.42 ± 3.31
Flavedo	0.971 ± 0.34	0.063 ± 0.012	0.14 ± 0.06 *	40.41 ± 10.15

Values are expressed as mean ± standard error of the mean (*n* = 3). * *p* < 0.01 DPPH: 2,2-Diphenyl-1-picrylhydrazyl; ABTS: 2,2′-Azino-*bis*(3-ethylbenzothiazoline-6-sulfonic acid).

## Data Availability

The original publication, which contains comprehensive research findings and detailed analyses, will be made available on the University of Urbino’s official website. Those who are keen on this research can access it at the following address: https://ora.uniurb.it/ (accessed from 1 January 2025). This platform ensures that the publication is easily accessible to students, researchers, and any other reader, fostering a broader dissemination of knowledge and facilitating academic engagement.

## Supplementary Materials

The following supporting information can be downloaded at: https://www.mdpi.com/article/10.3390/biom14101275/s1, Table S1: Scores and information from a BLAST search using as query the ITS sequence of *Citrus medica* var *Limoncella* [54,55]; Figure S1: Phylogenetic tree of selected *Citrus species*; Table S2: HPLC–MS/MS acquisition parameters (dynamic-MRM mode) used for the analysis of the 38 marker compounds; Figure S2: Original electrophoresis gels (A–D) used in DNA nicking assay for the albedo extract; Figure S3: Original electrophoresis gels (A–D) used in DNA nicking assay for the flavedo extract.
