# Supplementary material for: Morphological Characterization, Polyphenolic Profile, and Bioactive Properties of Limoncella, an Ancient Mediterranean Variety of Sweet Citrus
Source: Biomolecules. 2024 Oct 10;14(10):1275. doi: 10.3390/biom14101275 (PMC11505904; doi:10.3390/biom14101275)
Supplement: Supplementary file 1 [file biomolecules-14-01275-s001.zip › biomolecules-3229132-supplementary_materials.pdf]

biomolecules

MDPI

Supplementary Materials

Morphological Characterization, Polyphenolic Profile, and Bioactive Properties of *Limoncella*, an Ancient Mediterranean Variety of Sweet Citrus

Lucia Potenza[1^], Roberta Saltarelli[1^], Francesco Palma[1*], Laura Di Patria[1], Giosuè Annibalini[1], Sabrina Burattini[1], Pietro Gobbi[1], Laura Valentini[1], Giovanni Caprioli[2], Agnese Santanatoglia[2], Sauro Vittori[2], Elena Barbieri[1]

[1] Department of Biomolecular Sciences, University of Urbino Carlo Bo, 61029 Urbino, Italy
[2] Chemistry Interdisciplinary Project (CHip), School of Pharmacy, University of Camerino, 62032 Camerino, Italy
^Co-authorship
*Corresponding author

1. BLAST analysis of ITS sequence.

BLAST analysis was performed online in the NCBI site (https://blast.ncbi.nlm.nih.gov/) using as query sequence the ITS of Citrus medica var Limoncella (GenBank accession number: PQ149946) versus the database nucleotide col-lection (nr/nt), consists of GenBank+EMBL+DDBJ+PDB+RefSeq sequences, and selecting the organism Citrus (taxid:2706). The program selected was Megablast [1,2].

The results show the highest Total Score with the lowest E-value for the *Cirus medica* species.

Table S1 reports statistical values and information on nine selected ITS sequences of Citrus species compared with the obtained ITS sequence.

References

1. Morgulis, A.; Coulouris, G.; Raytselis, Y.; Madden, T.L.; Agarwala, R.; Schäffer, A.A. Database Indexing for Production MegaBLAST Searches. *Bioinformatics* 2008, *24*, 1757–1764, doi:10.1093/BIOINFORMATICS/BTN322.
2. Zhang, Z.; Schwartz, S.; Wagner, L.; Miller, W. A Greedy Algorithm for Aligning DNA Sequences. *J Comput Biol* 2000, *7*, 203–214, doi:10.1089/10665270050081478.

biomolecules

MDPI

Table S1: Scores and information from a BLAST search using as query the ITS sequence of *Citrus medica* var Limoncella

Description	Scientific Name	Accession	Total Score^	Query Cover	E-value*	% identity	Accession Length
Citrus medica 18S small subunit ribosomal RNA gene, internal transcribed spacer 1, 5.8S ribosomal RNA gene, internal transcribed spacer 2, and 26S large subunit ribosomal RNA gene, complete sequence	*Citrus medica*	ON479680.1	1129	100%	0	99.68%	7684
Citrus medica cultivar Indonesia small subunit ribosomal RNA gene, partial sequence; internal transcribed spacer 1, 5.8S ribosomal RNA gene, and internal transcribed spacer 2, complete sequence; and large subunit ribosomal RNA gene, partial sequence	*Citrus medica*	OL774794.1	1129	100%	0	99.68%	714
Citrus x aurantiifolia clone 6 small subunit ribosomal RNA gene, partial sequence; internal transcribed spacer 1, 5.8S ribosomal RNA gene, and internal transcribed spacer 2, complete sequence; and large subunit ribosomal RNA gene, partial sequence	*Citrus x aurantiifolia*	MF988685.1	1123	100%	0	99.51%	706
Citrus x aurantiifolia clone 4 internal transcribed spacer 1, partial sequence; 5.8S ribosomal RNA gene and internal transcribed spacer 2, complete sequence; and large subunit ribosomal RNA gene, partial sequence	*Citrus x aurantiifolia*	MF974187.1	1123	100%	0	99.51%	689
Citrus jambhiri 18S ribosomal RNA gene, partial sequence; internal transcribed spacer 1, 5.8S ribosomal RNA gene, and internal transcribed spacer 2, complete sequence; and 28S ribosomal RNA gene, partial sequence	*Citrus jambhiri*	JN681159.1	1123	100%	0	99.51%	700
Citrus limon voucher PS1609MT01 18S ribosomal RNA gene, partial sequence; internal transcribed spacer 1, 5.8S ribosomal RNA gene, and internal transcribed spacer 2, complete sequence; and 28S ribosomal RNA gene, partial sequence	*Citrus x limon*	FJ980439.1	1118	100%	0	99.35%	672
Citrus limon isolate CLIM-L1 internal transcribed spacer 1, partial sequence; 5.8S ribosomal RNA gene, complete sequence; and internal transcribed spacer 2, partial sequence	*Citrus x limon*	GQ225862.1	1050	93%	0	99.65%	575

Citrus megaloxycarpa isolate CMEG-S1 internal transcribed spacer 1, partial sequence; 5.8S ribosomal RNA gene, complete sequence; and internal transcribed spacer 2, partial sequence	*Citrus megaloxycarpa*	GQ225857.1	1029	91%	0	99.65%	564
Citrus indica isolate CIND-N25 internal transcribed spacer 1, partial sequence; 5.8S ribosomal RNA gene, complete sequence; and internal transcribed spacer 2, partial sequence	*Citrus indica*	GQ225846.1	1016	98%	0	96.72%	610

^Total score: Sum of the bit scores for all aligned regions between the query and the database sequence.
***E-value:** Expectation value, statistical value of likely, lower value means significant match.

biomolecules

MDPI

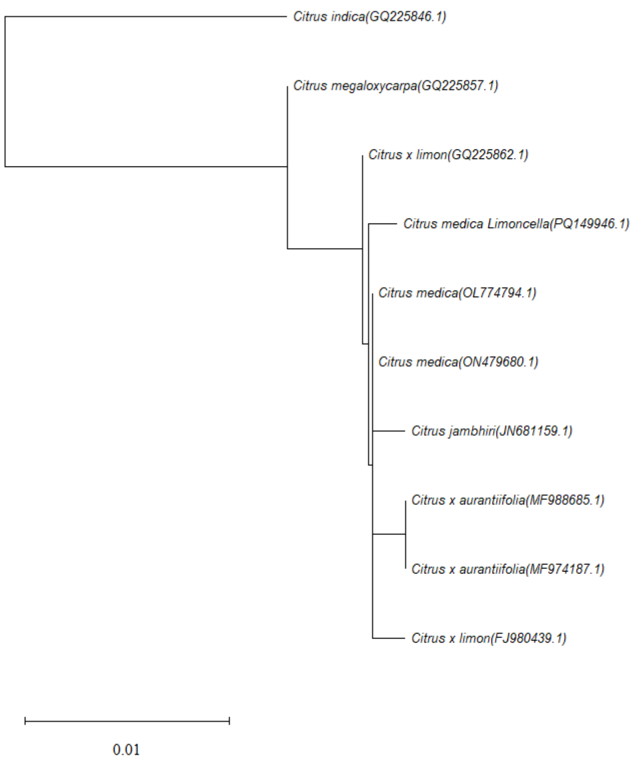

Figure S1: Phylogenetic tree of selected *Citrus* species constructed using the Neighbor-Joining method. The tree was generated with a Maximum Sequence Difference of 0.75. Branches are labeled using the format "Taxonomic Name (Sequence ID)," which allows identification of the species and corresponding sequence in Table S1. The topology represents evolutionary relationships among *Citrus* species, with branch lengths proportional to the genetic differences between sequences.

2. HPLC–MS/MS acquisition parameters

Table S2: HPLC–MS/MS acquisition parameters (dynamic-MRM mode) used for the analysis of the 38 marker compounds.

No.	Compounds	Precursor ion, *m/z*	Production, *m/z*	FR, V	CE, V	Polarity	Rt, min	Δ Rt
1	Gallic acid	169	125.2*	97	12	Negative	6.96	2
2	Neochlorogenic acid	353	191.2*, 179	82	12, 12	Negative	9.52	2
3	Delphindin-3-galactoside	465.01	303*	121	20	Positive	11.36	2
4	(+)-Catechin	289	245.2*,109.2	131	8, 20	Negative	11.44	2
5	Procyanidin B2	576.99	576.99*, 321.2	160	0, 32	Negative	12.41	2
6	Chlorogenic acid	353	191.2*, 127.5	82	12, 20	Negative	12.42	2
7	*p*-Hydroxybenzoic acid	137	93.2*	92	16	Negative	12.86	2
8	(-)-Epicatechin	289	245.1*, 109.1	126	8, 20	Negative	13.03	2
9	Cyanidin-3-glucoside	449	287.3*, 255.6	121	20, 20	Positive	13.14	2
10	Petunidin-3-glucoside	479.01	317*, 302	121	20, 44	Positive	13.26	2
11	3-Hydroxybenzoic acid	137	93.2*	88	8	Negative	13.59	2
12	Caffeic acid	179	135.2*, 134.1	92	12, 24	Negative	13.65	2
13	Vanillic acid	167	152.4*, 108.1	88	12, 20	Negative	14.32	2
14	Resveratrol	227	185*	131	12	Negative	14.40	2
15	Pelargonidin-3-glucoside	433.01	271*, 121	116	24, 50	Positive	14.52	2
16	Pelagonidin-3-rutinoside	579.01	271*	145	32	Positive	14.56	2
17	Malvidin-3-galactoside	493.01	331*, 315.1	121	20, 50	Positive	14.64	2
18	Syringic acid	196.9	182.2*, 121.2	93	8, 12	Negative	15.28	2
19	Procyanidin A2	575	575*, 285	170	0, 20	Negative	16.18	2
20	*p*-Coumaric acid	163	119.2*, 93.2	83	12, 36	Negative	16.70	2
21	Ferulic acid	193	134.2*, 131.6	83	12, 8	Negative	17.10	2
22	3,5-Dicaffeoylquinic acid	514.9	353.1*, 191	117	8, 28	Negative	17.61	2
23	Rutin	609	300.2*, 271.2	170	32, 50	Negative	17.73	2
24	Hyperoside	465.01	303*, 61.1	97	8, 50	Positive	18.33	2
25	Isoquercitrin	463	271.2*, 300.2	155	44, 24	Negative	18.36	2
26	Delphindin-3,5-digluco-side	462.9	300.1*	165	24	Negative	18.38	2
27	Phloridzin	435.39	273*, 167	155	8, 28	Negative	18.83	2
28	Quercitrin	446.99	300.2*, 301.2	160	24, 16	Negative	19.61	2
29	Myricetin	316.99	179.1*, 182	150	16, 24	Negative	19.61	2
30	Naringin	578.99	271.3*, 151.3	170	32, 44	Negative	19.62	2
31	Kaempferol-3-glucoside	447	284.2*, 255.2	170	24, 40	Negative	19.77	2
32	Hesperidin	611.01	303*, 334.8	112	20, 12	Positive	20.19	2
33	Ellagic acid	301	301*, 229	170	0, 24	Negative	21.41	2
34	*trans*-cinnamic acid	149	131.2	74	4	Positive	21.44	2
35	Quercetin	300.99	151.2*, 179.2	145	16, 12	Negative	21.87	2
36	Phloretin	272.99	167*, 123	116	8, 20	Negative	22.30	2
37	Kaempferol	287.01	153*, 69.1	60	36, 50	Positive	23.84	2
38	Isorhamnetin	314.99	300.2*, 196.1	145	16, 4	Negative	24.57	2

* These product ions were used for quantification.

FR., Fragmentor; CE, Collision energy; Rt, Retention time; ΔRt, Delta retention time

A

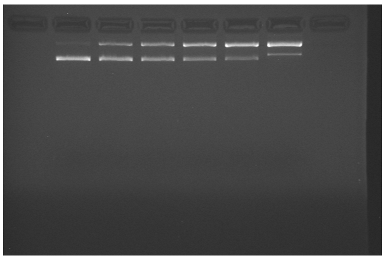

B

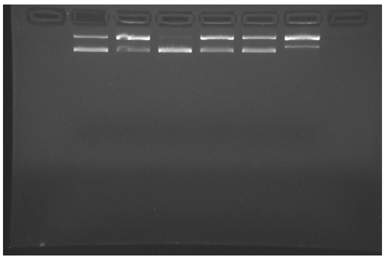

C

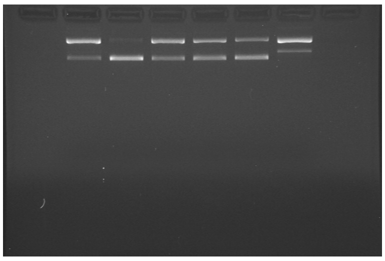

D

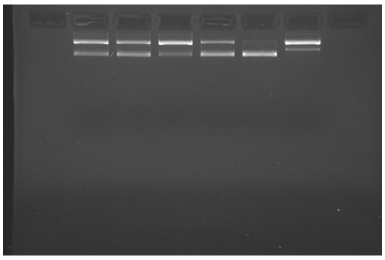

Figure S2: Original electrophoresis gels (A-D) used in DNA nicking assay for the albedo extract.

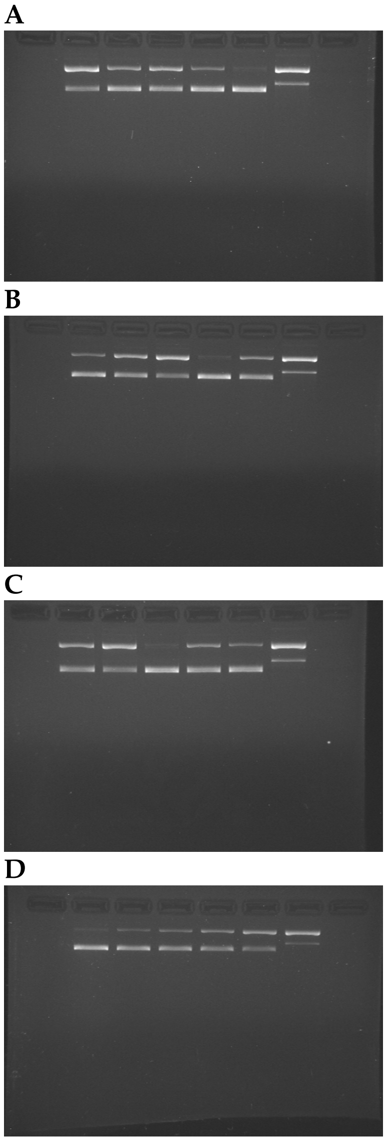

Figure S3: Original electrophoresis gels (**A-D**) used in DNA nicking assay for the flavedo extract.
